# Di-*tert*-butyl 2,2′-[(biphenyl-4,4′-diyl)­dioxy]diacetate

**DOI:** 10.1107/S1600536810023391

**Published:** 2010-06-23

**Authors:** Qamar Ali, Sammer Yousuf, Muhammad Raza Shah, Seik Weng Ng

**Affiliations:** a H.E.J. Research Institute of Chemistry, International Center for Chemical and Biological Sciences, University of Karachi, Karachi 75270, Pakistan; b Department of Chemistry, University of Malaya, 50603 Kuala Lumpur, Malaysia

## Abstract

The complete molecule of the title compound, C_24_H_30_O_6_, is generated by a crystallographic inversion centre. In the unique part of the mol­ecule, the four-atom –O–CH_2_–C(= O)–O– chain between the benzene ring and the *tert*-butyl group assumes a zigzag conformation [O—C—C—O torsion angle = −162.3 (1)°].

## Related literature

For a related structure, see: Shah *et al.* (2010 ▶).
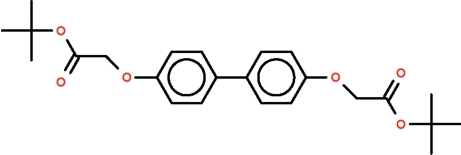


## Experimental

### 

#### Crystal data

C_24_H_30_O_6_
*M* *_r_* = 414.48
Monoclinic, 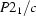

*a* = 9.9390 (7) Å
*b* = 12.6247 (8) Å
*c* = 9.8458 (7) Å
β = 114.645 (1)°
*V* = 1122.88 (13) Å^3^
*Z* = 2
Mo *K*α radiation
μ = 0.09 mm^−1^
*T* = 293 K
0.4 × 0.3 × 0.2 mm

#### Data collection

Bruker SMART APEX diffractometer
7450 measured reflections
2572 independent reflections
2029 reflections with *I* > 2σ(*I*)
*R*_int_ = 0.024

#### Refinement

*R*[*F* ^2^ > 2σ(*F* ^2^)] = 0.041
*wR*(*F* ^2^) = 0.120
*S* = 1.02
2572 reflections
136 parameters
H-atom parameters constrained
Δρ_max_ = 0.16 e Å^−3^
Δρ_min_ = −0.14 e Å^−3^

### 

Data collection: *SMART* (Bruker, 2002 ▶); cell refinement: *SAINT* (Bruker, 2002 ▶); data reduction: *SAINT*; program(s) used to solve structure: *SHELXS97* (Sheldrick, 2008 ▶); program(s) used to refine structure: *SHELXL97* (Sheldrick, 2008 ▶); molecular graphics: *X-SEED* (Barbour, 2001 ▶); software used to prepare material for publication: *publCIF* (Westrip, 2010 ▶).

## Supplementary Material

Crystal structure: contains datablocks global, I. DOI: 10.1107/S1600536810023391/lh5072sup1.cif

Structure factors: contains datablocks I. DOI: 10.1107/S1600536810023391/lh5072Isup2.hkl

Additional supplementary materials:  crystallographic information; 3D view; checkCIF report

## Figures and Tables

**Fig. 1.**
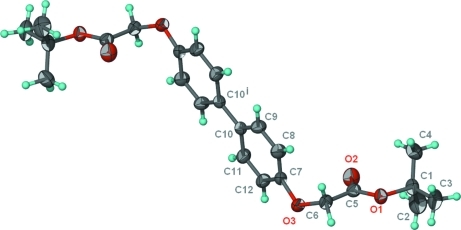
Thermal ellipsoid plot (Barbour, 2001) of C24H30O6 at the 50% probability level; hydrogen atoms are drawn as spheres of arbitrary radius. Symmetry code: (i) = 1 - x, 1 - y, 1 - z.

## supplementary crystallographic information

### Comment

We are interested in the solid-state structures of *V*-shaped molecules; recently we reported the crystal structure of 9,9-bis[4-(*tert*-butoxycarbonylmethyloxy)phenyl]fluorene (Shah *et al.*, 2010). For such a shape, the number of carbon atoms making up the kink must be an odd number. In the present compound, the two aromatic rings are directly connected; the molecule lies on a center of symmetry (Fig. 1). The four-atom –O–CH_2_–C(═ O)–O– chain between the aromatic ring and the *tert*-butyl group assumes a zigzag conformation [O–C–C–O torsion angle 162.3 (1) °].

### Experimental

4,4'-Dihydroxybiphenyl (1 g, 2.4 mmol) was dissolved in acetone (25 ml). To the solution was added potassium carbonate (0.67 g, 4.8 mmol) and *t*-butyl bromoacetate (0.75 ml, 4.8 mmol). The mixture was stirred at room temperature for 3 h. The solvent was evaporated under reduced pressure and the residue was dissolved in a mixture of water (50 ml) and dichloromethane (50 ml). The aqueous layer was extracted three times with dichloromethane. The combined organic phases were evaporated under reduced pressure and the solid material was recrystallized from *n*-hexane.

### Refinement

H-atoms were placed in calculated positions [C–H 0.93–0.97 Å, *U*(H) 1.2–1.5*U*(C)] and were included in the refinement in the riding model approximation.

### Crystal data

**Table:**

C_24_H_30_O_6_	*F*(000) = 444
*M**_r_* = 414.48	*D*_x_ = 1.226 Mg m^−^^3^
Monoclinic, *P*2_1_/*c*	Mo *K*α radiation, λ = 0.71073 Å
Hall symbol: -P 2ybc	Cell parameters from 2683 reflections
*a* = 9.9390 (7) Å	θ = 2.8–28.4°
*b* = 12.6247 (8) Å	µ = 0.09 mm^−^^1^
*c* = 9.8458 (7) Å	*T* = 293 K
β = 114.645 (1)°	Block, colorless
*V* = 1122.88 (13) Å^3^	0.4 × 0.3 × 0.2 mm
*Z* = 2	

### Data collection

**Table:**

Bruker SMART APEX diffractometer	2029 reflections with *I* > 2σ(*I*)
Radiation source: fine-focus sealed tube	*R*_int_ = 0.024
graphite	θ_max_ = 27.5°, θ_min_ = 2.3°
ω scans	*h* = −9→12
7450 measured reflections	*k* = −15→16
2572 independent reflections	*l* = −12→12

### Refinement

**Table:**

Refinement on *F*^2^	Primary atom site location: structure-invariant direct methods
Least-squares matrix: full	Secondary atom site location: difference Fourier map
*R*[*F*^2^ > 2σ(*F*^2^)] = 0.041	Hydrogen site location: inferred from neighbouring sites
*wR*(*F*^2^) = 0.120	H-atom parameters constrained
*S* = 1.02	*w* = 1/[σ^2^(*F*_o_^2^) + (0.0597*P*)^2^ + 0.1713*P*] where *P* = (*F*_o_^2^ + 2*F*_c_^2^)/3
2572 reflections	(Δ/σ)_max_ = 0.001
136 parameters	Δρ_max_ = 0.16 e Å^−^^3^
0 restraints	Δρ_min_ = −0.14 e Å^−^^3^

### Fractional atomic coordinates and isotropic or equivalent isotropic displacement parameters (Å^2^)

**Table:**

	*x*	*y*	*z*	*U*_iso_*/*U*_eq_	
O1	0.16964 (10)	1.03985 (7)	0.42919 (10)	0.0453 (2)	
O2	0.24672 (13)	0.91302 (9)	0.31464 (12)	0.0643 (3)	
O3	0.49943 (10)	0.88751 (7)	0.57432 (12)	0.0538 (3)	
C1	0.03883 (16)	1.07454 (11)	0.29402 (15)	0.0507 (4)	
C2	0.0877 (2)	1.11440 (17)	0.1761 (2)	0.0784 (5)	
H2A	0.1256	1.0563	0.1397	0.118*	
H2B	0.1636	1.1669	0.2193	0.118*	
H2C	0.0047	1.1452	0.0949	0.118*	
C3	−0.0216 (2)	1.16473 (14)	0.3541 (2)	0.0726 (5)	
H3A	−0.0548	1.1376	0.4260	0.109*	
H3B	−0.1032	1.1973	0.2733	0.109*	
H3C	0.0548	1.2163	0.4010	0.109*	
C4	−0.07095 (18)	0.98421 (15)	0.2398 (2)	0.0708 (5)	
H4A	−0.0985	0.9629	0.3184	0.106*	
H4B	−0.0266	0.9255	0.2119	0.106*	
H4C	−0.1574	1.0070	0.1549	0.106*	
C5	0.25888 (15)	0.96225 (10)	0.42366 (15)	0.0434 (3)	
C6	0.37853 (15)	0.94408 (10)	0.57882 (16)	0.0474 (3)	
H6A	0.4129	1.0118	0.6274	0.057*	
H6B	0.3377	0.9046	0.6374	0.057*	
C7	0.48857 (14)	0.77893 (10)	0.55146 (14)	0.0430 (3)	
C8	0.40501 (16)	0.71184 (11)	0.59620 (17)	0.0501 (3)	
H8	0.3454	0.7391	0.6398	0.060*	
C9	0.41070 (15)	0.60337 (10)	0.57559 (15)	0.0465 (3)	
H9	0.3543	0.5589	0.6066	0.056*	
C10	0.49740 (13)	0.55858 (9)	0.51049 (13)	0.0372 (3)	
C11	0.58025 (16)	0.62869 (11)	0.46692 (16)	0.0485 (3)	
H11	0.6399	0.6020	0.4230	0.058*	
C12	0.57629 (16)	0.73697 (11)	0.48722 (17)	0.0513 (4)	
H12	0.6332	0.7817	0.4573	0.062*	

### Atomic displacement parameters (Å^2^)

**Table:**

	*U*^11^	*U*^22^	*U*^33^	*U*^12^	*U*^13^	*U*^23^
O1	0.0429 (5)	0.0464 (5)	0.0447 (5)	0.0080 (4)	0.0165 (4)	−0.0013 (4)
O2	0.0713 (7)	0.0657 (7)	0.0547 (6)	0.0123 (5)	0.0250 (5)	−0.0130 (5)
O3	0.0412 (5)	0.0355 (5)	0.0814 (7)	0.0021 (4)	0.0224 (5)	−0.0019 (4)
C1	0.0454 (7)	0.0560 (8)	0.0483 (7)	0.0088 (6)	0.0171 (6)	0.0094 (6)
C2	0.0807 (12)	0.0946 (14)	0.0670 (11)	0.0121 (10)	0.0378 (10)	0.0271 (9)
C3	0.0677 (11)	0.0677 (11)	0.0821 (11)	0.0265 (9)	0.0309 (9)	0.0127 (9)
C4	0.0509 (9)	0.0787 (11)	0.0664 (10)	−0.0030 (8)	0.0082 (8)	0.0053 (8)
C5	0.0447 (7)	0.0381 (6)	0.0503 (7)	−0.0006 (5)	0.0227 (6)	−0.0037 (5)
C6	0.0470 (7)	0.0381 (6)	0.0538 (8)	0.0051 (5)	0.0177 (6)	−0.0026 (5)
C7	0.0361 (6)	0.0364 (6)	0.0503 (7)	0.0024 (5)	0.0117 (5)	0.0010 (5)
C8	0.0493 (8)	0.0448 (7)	0.0637 (8)	−0.0005 (6)	0.0312 (7)	−0.0061 (6)
C9	0.0489 (7)	0.0417 (7)	0.0549 (8)	−0.0054 (6)	0.0277 (6)	−0.0014 (5)
C10	0.0341 (6)	0.0379 (6)	0.0359 (6)	0.0010 (5)	0.0109 (5)	0.0033 (5)
C11	0.0487 (7)	0.0415 (7)	0.0645 (8)	0.0046 (6)	0.0327 (7)	0.0058 (6)
C12	0.0485 (8)	0.0399 (7)	0.0715 (9)	0.0004 (6)	0.0310 (7)	0.0085 (6)

### Geometric parameters (Å, °)

**Table:**

O1—C5	1.3379 (15)	C4—H4C	0.9600
O1—C1	1.4867 (16)	C5—C6	1.5118 (19)
O2—C5	1.2020 (16)	C6—H6A	0.9700
O3—C7	1.3861 (15)	C6—H6B	0.9700
O3—C6	1.4143 (16)	C7—C12	1.3787 (19)
C1—C4	1.513 (2)	C7—C8	1.3807 (19)
C1—C2	1.519 (2)	C8—C9	1.3890 (19)
C1—C3	1.518 (2)	C8—H8	0.9300
C2—H2A	0.9600	C9—C10	1.3905 (18)
C2—H2B	0.9600	C9—H9	0.9300
C2—H2C	0.9600	C10—C11	1.3929 (18)
C3—H3A	0.9600	C10—C10^i^	1.497 (2)
C3—H3B	0.9600	C11—C12	1.3845 (19)
C3—H3C	0.9600	C11—H11	0.9300
C4—H4A	0.9600	C12—H12	0.9300
C4—H4B	0.9600		
			
C5—O1—C1	121.76 (10)	O2—C5—C6	124.59 (12)
C7—O3—C6	119.66 (10)	O1—C5—C6	108.99 (10)
O1—C1—C4	109.05 (11)	O3—C6—C5	111.44 (11)
O1—C1—C2	110.05 (12)	O3—C6—H6A	109.3
C4—C1—C2	113.18 (14)	C5—C6—H6A	109.3
O1—C1—C3	102.28 (12)	O3—C6—H6B	109.3
C4—C1—C3	110.99 (14)	C5—C6—H6B	109.3
C2—C1—C3	110.73 (14)	H6A—C6—H6B	108.0
C1—C2—H2A	109.5	C12—C7—C8	119.36 (12)
C1—C2—H2B	109.5	C12—C7—O3	115.67 (12)
H2A—C2—H2B	109.5	C8—C7—O3	124.85 (12)
C1—C2—H2C	109.5	C7—C8—C9	119.45 (13)
H2A—C2—H2C	109.5	C7—C8—H8	120.3
H2B—C2—H2C	109.5	C9—C8—H8	120.3
C1—C3—H3A	109.5	C8—C9—C10	122.62 (12)
C1—C3—H3B	109.5	C8—C9—H9	118.7
H3A—C3—H3B	109.5	C10—C9—H9	118.7
C1—C3—H3C	109.5	C9—C10—C11	116.31 (11)
H3A—C3—H3C	109.5	C9—C10—C10^i^	121.94 (14)
H3B—C3—H3C	109.5	C11—C10—C10^i^	121.75 (14)
C1—C4—H4A	109.5	C12—C11—C10	121.79 (13)
C1—C4—H4B	109.5	C12—C11—H11	119.1
H4A—C4—H4B	109.5	C10—C11—H11	119.1
C1—C4—H4C	109.5	C7—C12—C11	120.47 (13)
H4A—C4—H4C	109.5	C7—C12—H12	119.8
H4B—C4—H4C	109.5	C11—C12—H12	119.8
O2—C5—O1	126.41 (13)		
			
C5—O1—C1—C4	64.56 (16)	C12—C7—C8—C9	−0.1 (2)
C5—O1—C1—C2	−60.14 (17)	O3—C7—C8—C9	−175.79 (13)
C5—O1—C1—C3	−177.86 (12)	C7—C8—C9—C10	−0.3 (2)
C1—O1—C5—O2	0.4 (2)	C8—C9—C10—C11	0.3 (2)
C1—O1—C5—C6	−178.70 (11)	C8—C9—C10—C10^i^	179.82 (14)
C7—O3—C6—C5	−77.86 (15)	C9—C10—C11—C12	−0.1 (2)
O2—C5—C6—O3	18.60 (19)	C10^i^—C10—C11—C12	−179.55 (14)
O1—C5—C6—O3	−162.30 (10)	C8—C7—C12—C11	0.3 (2)
C6—O3—C7—C12	153.70 (12)	O3—C7—C12—C11	176.43 (12)
C6—O3—C7—C8	−30.43 (19)	C10—C11—C12—C7	−0.3 (2)

Symmetry codes: (i) −*x*+1, −*y*+1, −*z*+1.
